# Quality evaluation of the spatial characteristics of basic public services: a case study of people with disabilities in China between 2012 and 2021

**DOI:** 10.3389/fpubh.2025.1551235

**Published:** 2025-04-02

**Authors:** Dan Yuan, Jiapei Guo

**Affiliations:** School of Management, Nanjing Normal University of Special Education, Nanjing, China

**Keywords:** people with disabilities, basic public services, quality evaluation, spatial characteristics, China

## Abstract

**Background:**

Under the social development goal of realizing common wealth, scientific evaluation and revealing the changing law of the quality of basic public services for people with disabilities is of great significance in promoting the sustainable and healthy development of the cause of people with disabilities.

**Methods:**

This paper constructs a comprehensive evaluation index system of the quality of basic public services for people with disabilities including 20 indicators in 5 dimensions of rehabilitation, education, facilities, culture and sports, and law, and applies the entropy value method and Dagum’s Gini coefficient to measure and compare the quality of basic public services for people with disabilities at the national, regional, and provincial levels in the period of 2012–2021.

**Results:**

(1) the quality of basic public services for persons with disabilities in China has been steadily improving since 2012, but the overall differences have a tendency to gradually widen, and such differences mainly come from the contribution of inter-regional differences, especially the eastern-western differences. (2) With the exception of the Northeast, the quality index of basic public services for persons with disabilities in the three major regions has been on an upward trend, with the western region showing the fastest rate of improvement. The quality of basic public services for persons with disabilities is highest in the East, but intra-regional differences are the greatest. (3) There are significant spatial differences in the quality of different types of basic public services.

**Conclusion:**

From 2012 to 2021, the provinces with high scores in the quality of basic public services for people with disabilities in China were mainly located in the economically developed eastern coastal areas, while most provinces in the central and western regions have deficiencies in rehabilitation, education, and cultural and sports services. The quality of each service is mostly at a low level and the matching degree is low. Suggestions have been put forward to improve the quality of basic public services for people with disabilities, including strengthening integration with social needs and addressing shortcomings in regional development.

## 1 Introduction

The provision of basic public services for individuals with disabilities has been a longstanding focus for government authorities and scholars alike. In recent years, the Central Committee of the Communist Party of China (CPC), under the leadership of General Secretary Xi Jinping, has placed significant emphasis on advancing the well-being and inclusion of individuals with disabilities. Initiatives such as fostering “enabling conditions and a supportive environment to ensure the equal participation of individuals with disabilities in societal development” and the commitment to “building a moderately prosperous society in all respects, ensuring that no person with disabilities is left behind” underscore this priority. Moreover, the principle that “no one with disabilities should be excluded from the path to common prosperity” reflects a renewed phase in promoting the development of disability-related initiatives and policies.

In 2017, the State Council identified services for individuals with disabilities as a critical element within the framework for equalizing basic public services. This was articulated in the *13th Five-Year Plan for Promoting the Equalization of Basic Public Services*. Subsequently, the Fourth Plenary Session of the 19th CPC Central Committee explicitly underscored the necessity of strengthening the public service system, enhancing the availability of essential public services, and addressing deficiencies in these services, particularly for the most vulnerable populations. Targeted measures were emphasized to cater to these groups effectively. Despite these efforts, achieving equal access to basic public services for individuals with disabilities remains challenging due to their limited development capacity and low levels of social participation. In this context, a thorough evaluation of the current status of basic public services for people with disabilities is vital. Such an assessment will not only consolidate the achievements of building a moderately prosperous society but also serve as a cornerstone in China’s broader strategy to construct a modern socialist country. Addressing this issue is a pressing topic requiring scholarly attention.

The equitable provision of public services has long been a topic of academic and policy interest. Since the 19th century, Wagner has argued for the state’s responsibility in ensuring fair access to public services (1). Later, Di Ji and Samuelson expanded upon this concept by providing systematic definitions and analyses of public services’ attributes and functions (2, 3). In recent years, the relevant empirical research on basic public services has been more fully explored by academics. Most of the scholars’ studies have focused on the following three aspects: (1) Connotation analysis. Academics and politicians mainly focus on basic public services to carry out theoretical research and exploration, and qualitatively answer the connotation characteristics, goal orientation and realization path of the equalization of basic public services (4–6). It is considered that basic public service is to guarantee the basic needs for the survival and development of all people, which is the basic function of modern government (7), and the continuous promotion of its equalization process aims to guarantee that the public can obtain the most basic conditions for survival and development in a fair and accessible way, as well as the basic social conditions required for the realization of people’s all-round development (8). In terms of path selection, it is proposed to promote the transformation of the government’s ‘single-centre governance’ model to a ‘multi-centre governance’ model in which the government, the market and society cooperate, which is the optimal path to achieve the sharing of basic public services in the region (9). (2) Level measurement. Scholars have further expanded the research field to the construction of indicators and level measurement of basic public services in a broad sense (10, 11), and most of the research results are based on the construction of a comprehensive evaluation system from the dimensions of health care, public education, social security, culture and sports, infrastructure, etc. Some scholars have based their research on the indicators of employment, education, social security, culture and sports. Some scholars have also conducted comprehensive evaluation based on basic public services for persons with disabilities such as employment, education and sports, and explored their impact on the quality of life and economic and social development (12–18). In terms of research methodology, entropy analysis, DEA analysis, sequential logistic model, coupled coordination model, etc. are mostly used (19–22); the research scale is based on provincial, municipal and county areas (23–25); (3) Influencing factors. Currently, scholars mostly use linear regression models to explore the impact of financial system, management system, economic development, etc. on the equalization of basic public services, hierarchical linear models have studied the impact of pro-environmental behavior (26), system dynamic GMM methods to test the degree of financial ecological environment, economic and social factors of the equalization (27), and spatial econometric models to explore the relationship between basic public services and regional economic growth, urbanization development level, income distribution, and rural migrant workers’ willingness to integrate into cities (28–31).

The results of the current research show that basic public service development has received extensive academic attention, characterized by systematic and relatively in-depth exploration. However, there is still potential for further depth in several areas. In terms of research groups, the perspective of the able-bodied is often taken, which may have overlooked the unique physiological, psychological and social needs of people with disabilities; in terms of research perspectives and content, there are still fewer in-depth explorations of the spatio-temporal characteristics of basic public services for people with disabilities from a geographical perspective.

People with disabilities face significant barriers to accessing equal opportunities for development compared to the general population. They require diverse and specialized public services that address their unique needs. The construction of a disability service system is essential for promoting their holistic development. Such a system not only ensures the provision of basic living conditions but also fosters a sense of security, dignity, and respect. It mitigates the social and psychological challenges associated with disabilities, alleviates the pressures faced by individuals with disabilities and their families, and unlocks their potential. By motivating and empowering people with disabilities to participate fully in social life, this system enhances their quality of life and promotes harmonious integration into society.

Therefore, this paper takes 31 provinces and cities in China as the research unit, takes the group of certified persons with disabilities as the target population, and uses entropy value analysis and the Dagum Gini coefficient to measure the quality of basic public services for persons with disabilities in China from 2012 to 2021, to reveal the pattern of change in different spatial scales and the characteristics of regional differences in the hope of providing scientific support for the advancement of the construction of the equalization of basic public services for persons with disabilities in the new period.

## 2 Materials and methods

### 2.1 Construction of the indicator system

Owing to their physical limitations, persons with disabilities have different needs for public services in terms of the extent and content of their needs from those of able-bodied persons. In order to meet the special needs of persons with disabilities, the public sector, led by the Government, provides a range of public services, including rehabilitation, education, employment, culture, sports, facilities and law. Among them, rehabilitation services are the core, helping persons with disabilities to restore, improve or compensate for their functions; education is a key way for persons with disabilities to improve themselves and their ability to adapt to society; employment is the main breakthrough for persons with disabilities to enhance their self-worth and realize their economic independence; the construction of facilities breaks down the physical barriers for persons with disabilities to participate in social life and facilitates their participation in society; cultural and sports activities are important components of the spiritual enrichment of the disabled; and the law protects the rights and interests of persons with disabilities. The construction of facilities can break the physical barriers for the disabled to participate in social life and promote their social participation.

For this reason, based on the connotation of basic public services, taking into account the special characteristics of disabled people’s groups, and following the principles of scientific, systematic, operability and accessibility in the selection of indicators, and taking into account both quantitative and qualitative indicators, this paper constructs a system of indicators for evaluating the quality of China’s basic public services for the disabled, which contains five dimensions and 20 indicators, such as rehabilitation, education, facilities, culture and sports and law. Among them, indicators related to the five dimensions generally include: labor force, facilities, funding and effects.

Therefore, in the process of constructing the indicator system, the rehabilitation service dimension selected the density of rehabilitation institutions, the number of rehabilitation talents per 10,000 people, the number of coordinators per county and city carrying out community rehabilitation, and the number of rehabilitation services provided by each rehabilitation institution; the education service dimension selected the density of special education schools, the number of teachers with bachelor’s degree or above per special education school, the number of graduates of special education schools, and the area per student of special education schools. The education service dimension selects the density of special education schools, the number of teachers with bachelor’s degree or above in each special education school, the number of graduates of special education schools, and the floor space per capita of special education students; the facility dimension selects the total investment in per capita completed comprehensive service facilities for persons with disabilities, the percentage of the construction scale of the cumulative completed rehabilitation facilities for persons with disabilities, the percentage of the construction scale of the cumulative completed comprehensive service facilities for persons with disabilities, and the density of childcare service organisations; and the culture and sports dimension selects the number of competitions and exhibitions of cultural and artistic activities for persons with disabilities, the number of art troupes of persons with disabilities, and the density of sports training bases for persons with disabilities, The legal dimension selects the density of legal aid coordination organisations for persons with disabilities, the density of legal aid workstations for persons with disabilities, the number of legal publicity and education activities, and the number of participants per capita in legal publicity and education activities.

The assessment of the quality of basic public services for persons with disabilities is a comprehensive evaluation with multiple indicators. In order to avoid the influence of subjective judgement and ensure the accuracy and reliability of the results, this paper adopts entropy analysis, an objective assignment method, to determine the weights of the indicators (32) (Table 1). The criteria for determining the weights of the indicators in this method are mainly based on the concept of entropy in information theory, i.e., for a certain indicator, if the degree of dispersion of its observations is greater, i.e., the smaller the information entropy is, the more information the indicator provides, and the greater the impact on the comprehensive evaluation (weight), and vice versa, the smaller it is (33).

**Table 1:** Evaluation index system and weights for basic public services for individuals with disabilities in China.

System layer		Indicator layer	Weight
Basic public service	Rehabilitation service	Density of rehabilitation institutions	0.0507
		Number of rehabilitation professionals per 10,000 people	0.0491
		Number of coordinators in counties and cities conducting community rehabilitation	0.0506
		Number of rehabilitation services provided by each rehabilitation institution	0.0498
	Education service	Density of special education schools	0.0533
		Number of undergraduate and above teachers in each special education school	0.0499
		Number of graduates from special education schools	0.0473
		Per capita land area for students in special education schools	0.0515
	Facility service	Cumulative total investment in completed comprehensive facilities for people with disabilities	0.0552
		Proportion of completed rehabilitation facility construction for people with disabilities	0.0487
		Proportion of completed comprehensive facility construction for people with disabilities	0.055
		Density of care service institutions	0.0505
	Culture and sports service	Cultural and artistic competitions and exhibitions for people with disabilities	0.0504
		Number of artistic groups for people with disabilities	0.0544
		Density of sports training facilities for people with disabilities	0.0494
		Number of participants in physical fitness activities for people with disabilities	0.0465
	Legal service	Density of legal aid coordination organizations for people with disabilities	0.0497
		Density of legal aid workstations for people with disabilities	0.0499
		Public legal education activities	0.0465
		Number of participants in public legal education activities per capita	0.0417

### 2.2 Research methods

Although in the process of application, entropy value analysis has limitations such as strong dependence on samples and the inability to directly reflect the correlation of indicators, entropy value analysis, as an objective and precise multi-attribute decision-making analysis method, still demonstrates a strong application value and advantages in the fields of environmental evaluation, economic benefit analysis and assessment of the level of social development. Evaluation of the quality of basic public services for people with disabilities is a comprehensive evaluation work consisting of multi-dimensional and multi-indicators such as rehabilitation, education, facilities, culture, sports and law. In order to objectively evaluate the weight of each indicator within the subsystem and avoid the subjectivity of human judgment, this paper adopts entropy analysis, an objective assignment method (32, 33), to determine the weight of each indicator and calculate the basic public service quality index for people with disabilities.

Traditional indices, such as the Tyrell index and the Gini coefficient, often rely on assumptions of normal distribution and homoscedasticity. These assumptions limit their ability to account for overlaps between subgroup samples, making it difficult to decompose the indices into sub-components with clear economic interpretations (34). To address these limitations, Dagum (35) proposed a method that refines the decomposition of the Gini coefficient by accounting for sample overlaps. This innovation allows for a more nuanced analysis of disparities and their sources.

In this study, Dagum’s Gini coefficient decomposition is applied to examine regional disparities in the provision of basic public services for individuals with disabilities across China. The analysis focuses on four key regions: eastern, central, western, and northeastern China. The model is constructed as follows:


(1) $$G_{jh}=\frac{\sum_{i=1}^{n_{j}}\sum_{r=1}^{n_{h}}|y_{ji}-y_{\mathrm{hr}}|}{n_{j}n_{h}\left(\bar{y_{j}}-\bar{y_{h}}\right)}$$

(2) $$G=\overset{k}{\underset{j=1}{\sum }}G_{jj}p_{j}s_{j}+\overset{k}{\underset{j=1}{\sum }}\underset{h\neq j}{\sum }G_{jh}p_{j}s_{h}D_{jh}+\overset{k}{\underset{j=1}{\sum }}\underset{h\neq j}{\sum }G_{jh}p_{j}s_{h}\left(1-D_{jh}\right)$$

(3) $$G=G_{w}+G_{nb}+G_{t}$$

Equation 1 defines the between-group Gini coefficient ( G ) and Equation 2 defines the overall Gini coefficient (*G*), where:

-
k represents the number of regions, set to 4, corresponding to China’s regional divisions.
-
n denotes the total number of provinces, set to 31, representing the scope of the study.
-
$\frac{n_{j}}{n_{h}}$ is the ratio of provinces in region j to those in region h .
-
$\frac{y_{ji}}{y_{\mathrm{hr}}}$ is the ratio of service quality scores for individuals with disabilities in the i -th province of region j to the r -th province of region h .
-
$\bar{y}$ refers to the national average score for the quality of basic public services for people with disabilities.

In Equation 3, the overall Gini coefficient ( G ) is decomposed into three components:

- Intra-regional variation ( $G_{w}$ ): reflects disparities within each of the four regions, capturing inequalities among provinces in the same region.
- Inter-regional variation ( $G_{nb}$ ): quantifies disparities between the four regions, providing insights into differences driven by regional factors, such as economic development, infrastructure, and public service implementation.
- Hypervariance density ( $G_{t}$ ): addresses cross-regional overlap in service quality scores, capturing the extent to which variations across provincial boundaries contribute to overall disparities. This component rectifies limitations of traditional methods, which often neglect such interactions.

By decomposing the Gini coefficient, this approach provides a detailed examination of the sources of disparities in the quality of basic public services for people with disabilities. It highlights the contributions of both intra-and inter-regional differences while accounting for overlapping effects.

This analytical framework offers valuable insights into the spatial distribution of service quality and identifies key areas requiring policy intervention. The findings can inform targeted measures to reduce inequalities and enhance the equity of basic public services for individuals with disabilities across China’s diverse regions.

### 2.3 Data

This study analyzes the provision of basic public services for individuals with disabilities across 31 provinces, autonomous regions, and municipalities directly governed by the central government in China during the period from 2012 to 2021. Taiwan, Hong Kong, and Macao are excluded from the analysis due to the unavailability of relevant data.

The comprehensive evaluation index system incorporates a wide range of indicators reflecting various dimensions of public services for individuals with disabilities. These include:

- Administrative and service coverage: area of administrative districts, the number of licensed individuals with disabilities.
- Rehabilitation services: The number of rehabilitation professionals, disability rehabilitation institutions, individuals receiving basic rehabilitation services, and social rehabilitation coordinators.
- Education services: special education schools, the number of teachers with a bachelor’s degree or higher in special education schools, special education school graduates, special education school enrollment, and coverage area of special education schools.
- Infrastructure and investment: comprehensive service facilities for individuals with disabilities, total investment in these facilities, and total investment in rehabilitation facilities.
- Cultural and recreational services: childcare service institutions, Braille and talking book libraries for the visually impaired in public libraries.
- Cultural and sports activities: cultural troupes for individuals with disabilities, sports training bases for persons with disabilities, and participation in mass sports and fitness activities.
- Legal assistance and advocacy: coordinating organizations for legal assistance, workstations for legal assistance, and participation in legal literacy and public education programs.

Data for these indicators are primarily sourced from the *China Statistical Yearbook on the Cause of Persons with Disabilities* (2012–2021), published by the National Bureau of Statistics of China. This dataset provides a comprehensive basis for evaluating the quality and distribution of public services for individuals with disabilities.

The regional classification into eastern, central, western, and northeastern regions follows the methodology established by the *National Bureau of Statistics* in 2011. This classification facilitates a comparative analysis of disparities and development patterns across China’s major regions.

## 3 Results

### 3.1 Nationwide quality and evolution of basic public services for individuals with disabilities

Using Entropy Analysis and the Dagum Gini coefficient, this study calculated the comprehensive quality index for basic public services for individuals with disabilities, along with sub-dimension scores at the national, regional, and provincial levels. Since 2012, the Chinese government has demonstrated a strong commitment to enhancing these services through increased financial investment and policy initiatives. By 2021, national financial subsidies for disability services reached 1.482 billion yuan, accounting for 0.0126% of total national financial expenditure. This sustained investment contributed to a significant improvement in the national quality index for basic public services for individuals with disabilities, which increased from 0.0578 in 2012 to 0.1368 in 2021 (Table 2).

**Table 2:** Quality index and sub-dimension scores of basic public services for individuals with disabilities in China, 2012–2021.

Year	Total score	Rehabilitation service	Education service	Facilities service	Culture and sports service	Legal service
2012	0.0578	0.0129	0.0132	0.0085	0.0116	0.0116
2013	0.0766	0.0158	0.0165	0.0148	0.0170	0.0125
2014	0.0869	0.0182	0.0206	0.0161	0.0182	0.0138
2015	0.0930	0.0175	0.0189	0.0173	0.0185	0.0207
2016	0.0889	0.0164	0.0196	0.0187	0.0154	0.0188
2017	0.1000	0.0184	0.0199	0.0212	0.0224	0.0181
2018	0.1098	0.0214	0.0208	0.0252	0.0238	0.0185
2019	0.1221	0.0239	0.0218	0.0280	0.0285	0.0199
2020	0.1282	0.0264	0.0240	0.0281	0.0226	0.0272
2021	0.1368	0.0293	0.0267	0.0315	0.0227	0.0266
2012–2015	0.0352	0.0046	0.0056	0.0088	0.0069	0.0092
2016–2021	0.0479	0.0129	0.0071	0.0128	0.0073	0.0078

In 2012, among the five dimensions, education services recorded the highest sub-dimension score (0.0132), followed by rehabilitation services (0.0129), cultural and sports services (0.0116), legal services (0.0116), and facility services (0.0085). By 2021, facility services achieved the highest score (0.0315), followed by rehabilitation services (0.0293), education services (0.0267), legal services (0.0266), and cultural and sports services (0.0227). Facility services and rehabilitation services exhibited the most substantial improvements during the 12th and 13th Five-Year Plan periods, with their combined contributions to the overall quality index reaching 29 and 21%, respectively. These findings underscore the prioritization of facility and rehabilitation services in improving the quality of public services for individuals with disabilities during this period.

The focus of public service development for individuals with disabilities has evolved over time, shaped by economic growth, social development, and national policies. Rehabilitation and education services, in particular, play a vital role in fostering societal integration for individuals with disabilities. Rehabilitation services are crucial for enhancing physical function and social adaptability, while education services empower individuals to achieve personal value and contribute to social progress (36).

During the 12th Five-Year Plan period, efforts to ensure “universal access to rehabilitation services” were intensified. Key measures included the implementation of specialized programs, the establishment of rehabilitation institutions, and the cultivation of a professional talent pool, which collectively resulted in 13 million individuals benefiting from rehabilitation services. In 2016, the *Basic Rehabilitation Service Directory for Persons with Disabilities* and the *Implementation Plan for Precision Rehabilitation Services for Persons with Disabilities* were introduced, further specifying the scope and proportional allocation of rehabilitation services.

Simultaneously, significant improvements in special education were observed. Between 2012 and 2021, the number of special education schools increased from 1,853 to 2,288, while the total land area expanded from 6.63 million to 12.29 million square meters. Student enrollment grew at an average annual rate of 10.36%, and the number of full-time teachers with bachelor’s degrees or higher increased by 9.82% annually. These advancements contributed to education and rehabilitation services maintaining the highest scores among the five dimensions during the study period.

**Figure 1:**
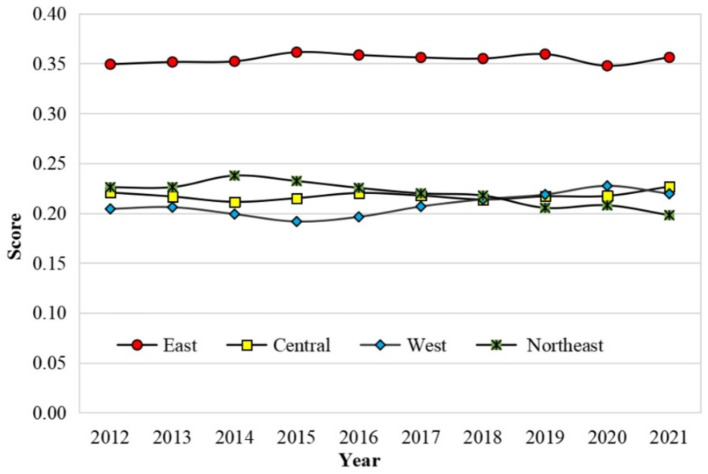
Trends in basic public services for persons with disabilities across four regions of China (2012–2021).

Facility services demonstrated the highest growth among all dimensions, with an average annual increase of 15.64%. This improvement aligns with the State Council’s *Guiding Opinions on Accelerating the Construction of the Social Security System and Service System* for Persons with Disabilities (2010), which emphasized the role of comprehensive service facilities, particularly care institutions, as critical infrastructure for public service delivery. Subsequent policy documents, such as the *13th Five-Year Plan for Accelerating the Well-Off Process for Persons with Disabilities and the Healthy China 2030 Plan*, further highlighted the importance of facility services in addressing the evolving needs of individuals with disabilities and supporting the modernization of disability services (Figure 1).

Conversely, cultural and sports services experienced the slowest growth during the study period, dropping from third place in 2012 to last place in 2021. This stagnation can be attributed to limited opportunities for self-directed activities, inadequate organizational support, and insufficient funding (37, 38).

Legal services ranked fourth in 2021 but exhibited the second-highest average annual growth rate after facility services. This trend reflects progress in establishing legal aid platforms and increasing the awareness and capacity of individuals with disabilities to safeguard their rights. The *Law of the People’s Republic of China on the Protection of Persons with Disabilities* (1991) provided a foundational legal framework for protecting the rights of individuals with disabilities. Between the 12th and 13th Five-Year Plan periods, seven five-year development plans were issued to further safeguard these rights. By 2021, the number of legal aid stations had increased to 2,845, and legal literacy and public education activities had expanded significantly, with participation rates rising by 48 times compared to 2012.

### 3.2 Regional comparison and changes in the quality of basic public services for individuals with disabilities

**Figure 2:**
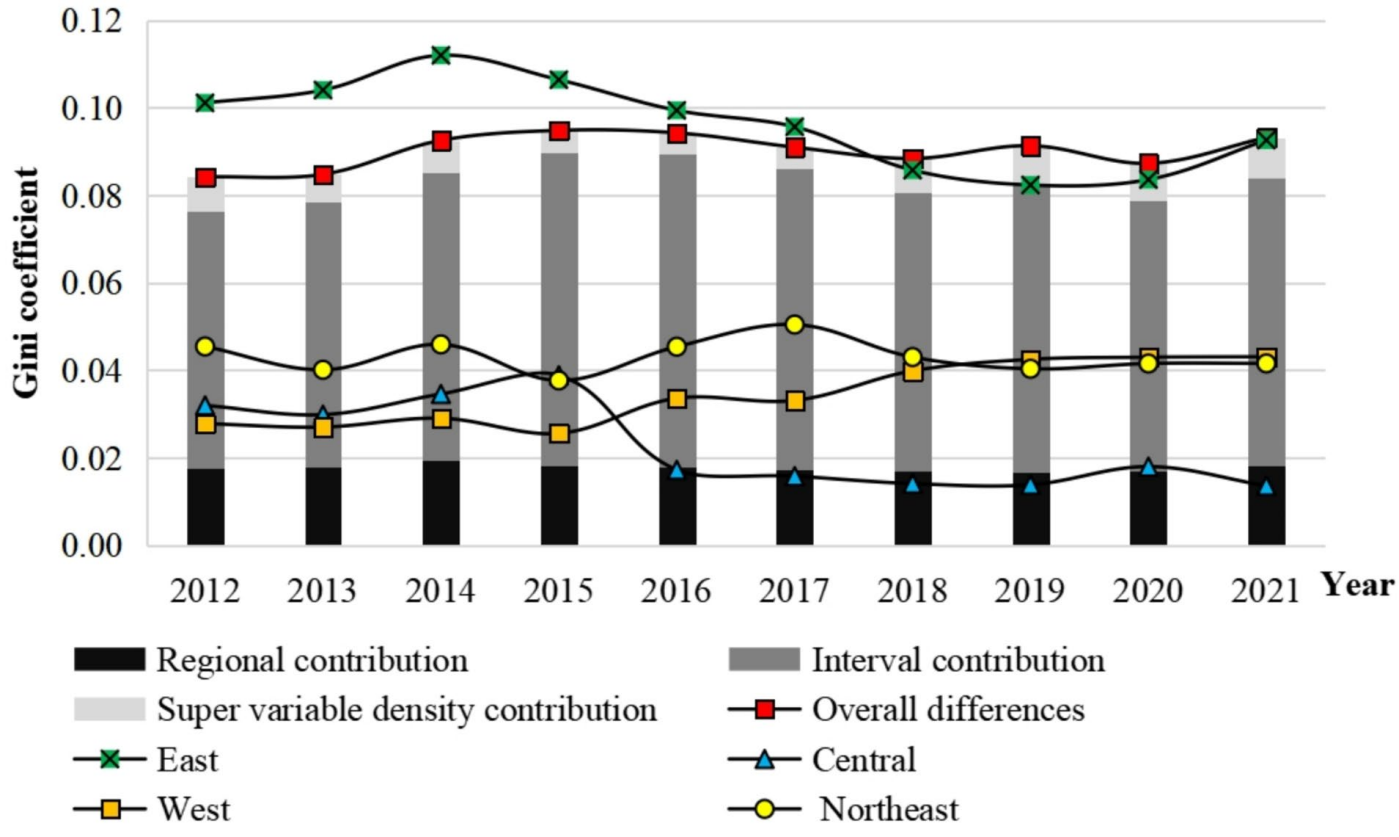
Evolution of basic public services for individuals with disabilities in China (2012–2021).

A comparison of the basic public service quality index for individuals with disabilities across China’s four major regions reveals significant regional disparities. In 2012, the eastern region recorded the highest quality index, followed by the central and northeastern regions, with the western region scoring the lowest. However, by 2019, the central and western regions surpassed the northeastern region in the quality index for basic public services for individuals with disabilities, driven by relatively faster rates of improvement in these regions (Figure 2).

From 2012 to 2021, the basic public service quality index for individuals with disabilities improved across all regions, with the exception of the northeastern region. The eastern, central, and western regions experienced increases in their indices from 0.3492, 0.2207, and 0.2042 to 0.3561, 0.2266, and 0.2194, respectively. Between 2012 and 2015, the eastern region demonstrated the most rapid improvement, followed by the northeastern region. However, during the period from 2016 to 2021, the western region exhibited the fastest improvement, followed by the central region. This trend indicates a narrowing of the quality gap in basic public services for individuals with disabilities among these regions.

Since the 18th National Congress of the Communist Party of China, there has been a heightened emphasis on coordinated regional development. The central government has implemented targeted poverty alleviation initiatives as part of its broader objective to establish a moderately prosperous society in all respects. By the end of 2020, many impoverished villages and registered disabled individuals in the central and western regions had risen above the poverty line according to national standards. These efforts have significantly contributed to the rapid development of services for individuals with disabilities in these regions.

In contrast, the northeastern region has seen comparatively limited improvement. Since 2014, the provinces of Heilongjiang, Jilin, and Liaoning have experienced economic growth rates below the national average, constraining their capacity to allocate resources for the development and construction of facilities for individuals with disabilities. Furthermore, the diminishing impact of revitalization strategies in the region has further exacerbated these challenges. Consequently, the quality of basic public services for individuals with disabilities in the northeastern region has shown the least improvement during the study period (39).

According to the scores from various dimensions (Table 3), the eastern region consistently achieved the highest scores in rehabilitation services, educational services, facility services, legal services, and cultural and sports services. Conversely, the western region recorded the lowest scores in educational, rehabilitation, and facility services, while the central region had the lowest score in cultural services, and the northeastern region ranked lowest in legal services.

**Table 3:** Changes in the scores of basic public service quality for individuals with disabilities across four major regions of China.

Year	Region	Rehabilitation service	Education service	Facilities service	Culture and sports service	Legal service
2012	East	0.0655	0.0711	0.0718	0.0729	0.0687
	Central	0.0531	0.0500	0.0354	0.0458	0.0438
	West	0.0385	0.0392	0.0343	0.0346	0.0485
	Northeast	0.0451	0.0506	0.0548	0.0401	0.0361
2015	East	0.0748	0.0688	0.0735	0.0738	0.0734
	Central	0.0457	0.0442	0.0351	0.0418	0.0447
	West	0.0376	0.0418	0.0359	0.0359	0.0349
	Northeast	0.0564	0.0511	0.0503	0.0427	0.0375
2021	East	0.0682	0.0706	0.0743	0.0739	0.0654
	Central	0.0480	0.0449	0.0389	0.0475	0.0602
	West	0.0439	0.0461	0.0489	0.0326	0.0357
	Northeast	0.0389	0.0445	0.0411	0.0394	0.0370
2012–2021	East	0.0027	−0.0005	0.0025	0.0010	−0.0033
	Central	−0.0051	−0.0052	0.0035	0.0018	0.0164
	West	0.0054	0.0068	0.0147	−0.0020	−0.0128
	Northeast	−0.0063	−0.0061	−0.0137	−0.0007	0.0009

The economically developed eastern region exerts a more significant influence on the development of services for people with disabilities due to its advanced urbanization processes. Improvements in public services for individuals with disabilities in this region are driven by a combination of external funding, consumption support, and internal demand fueled by regional development. As China transitions to addressing the imbalance between growing societal needs and uneven development, demand for cultural and sports activities among individuals with disabilities has surged, creating pressure for enhanced provision in these areas (40).

In comparison, the central, western, and northeastern regions exhibit substantial deficiencies in public service quality, particularly in facility, cultural and sports, and legal services. The northeastern region, in particular, suffers from fewer healthcare facilities and lower distribution density, attributed to natural conditions and slower economic development. Similarly, economic and financial constraints in the central and western regions limit investments in infrastructure, cultural and sports activities, and legal rights protection services.

Further analysis of the Gini coefficient for basic public service quality reveals trends in regional disparities (Figure 2). Overall, regional disparities in service quality for people with disabilities remained relatively low, with a mean value of 0.0902 during the study period. The Dagum Gini coefficient indicates an initial increase followed by a decline, suggesting progressive improvements in fairness and coordination in public service provision nationwide.

The average Gini coefficients for the four major regions’ service quality dimensions from 2012 to 2021 were as follows, in ascending order: Central region (0.0136), Northeast region (0.0416), Western region (0.0431), and Eastern region (0.0963). Except for occasional years in the eastern region, the Gini coefficients for the central, western, and northeastern regions consistently remained below the national average. This indicates that disparities within the central region were the smallest, while the eastern region exhibited the largest internal differences. The overall variation in service quality was predominantly driven by inter-regional disparities.

The relatively uniform economic development levels within the central region contributed to more equitable distribution of public services for individuals with disabilities. In contrast, the polarized development of provinces like Beijing, Shanghai, and Hainan within the eastern region exacerbated spatial imbalances in service quality. In the northeastern region, disparities remained relatively stable, but a decline in scores in Liaoning Province since 2017 slightly reduced internal differences.

In the western region, the Dagum Gini coefficient displayed a “decline-rise” trend. With the exception of 2016 and 2018, the coefficient exhibited a steady increase in most years, reflecting widening regional disparities. This was largely attributed to rapid improvements in provinces such as Sichuan, Chongqing, and Ningxia, which outpaced other provinces in the region.

The regional Gini coefficient contribution to the quality of basic public services for people with disabilities in China was 0.0176 at the beginning of the sample, followed by fluctuations, and finally increased to 0.0181 at the end of the study period. During the same period, the contribution of the interval Gini coefficient fluctuated from 0.0589 in the initial stage to 0.0659, with a change of over 11.8%, far higher than the contribution of the Gini coefficient within the region.

On the one hand, this indicates that interval differences are the main source of the overall differences in the quality index of basic public services for people with disabilities nationwide. Reducing regional disparities, especially between the eastern and western regions, is an important dimension for optimizing the layout of basic public services for people with disabilities nationwide and promoting equalization of basic public services for people with disabilities in the future. On the other hand, it also indicates that the positive cycle of high-level service supply, high-density facility layout, and high starting point coordinated development has played an important role in the eastern region, gradually widening the gap with other regions.

Meanwhile, because the inter regional hypervariable density reflects the contribution of the overlapping parts between provinces to the overall differences, its proportion in the overall differences is not high, which means that the division method of China’s four major regions can effectively distinguish different types of provinces and has strong rationality.

### 3.3 Provincial comparison of the quality of basic public services for individuals with disabilities

**Figure 3:**
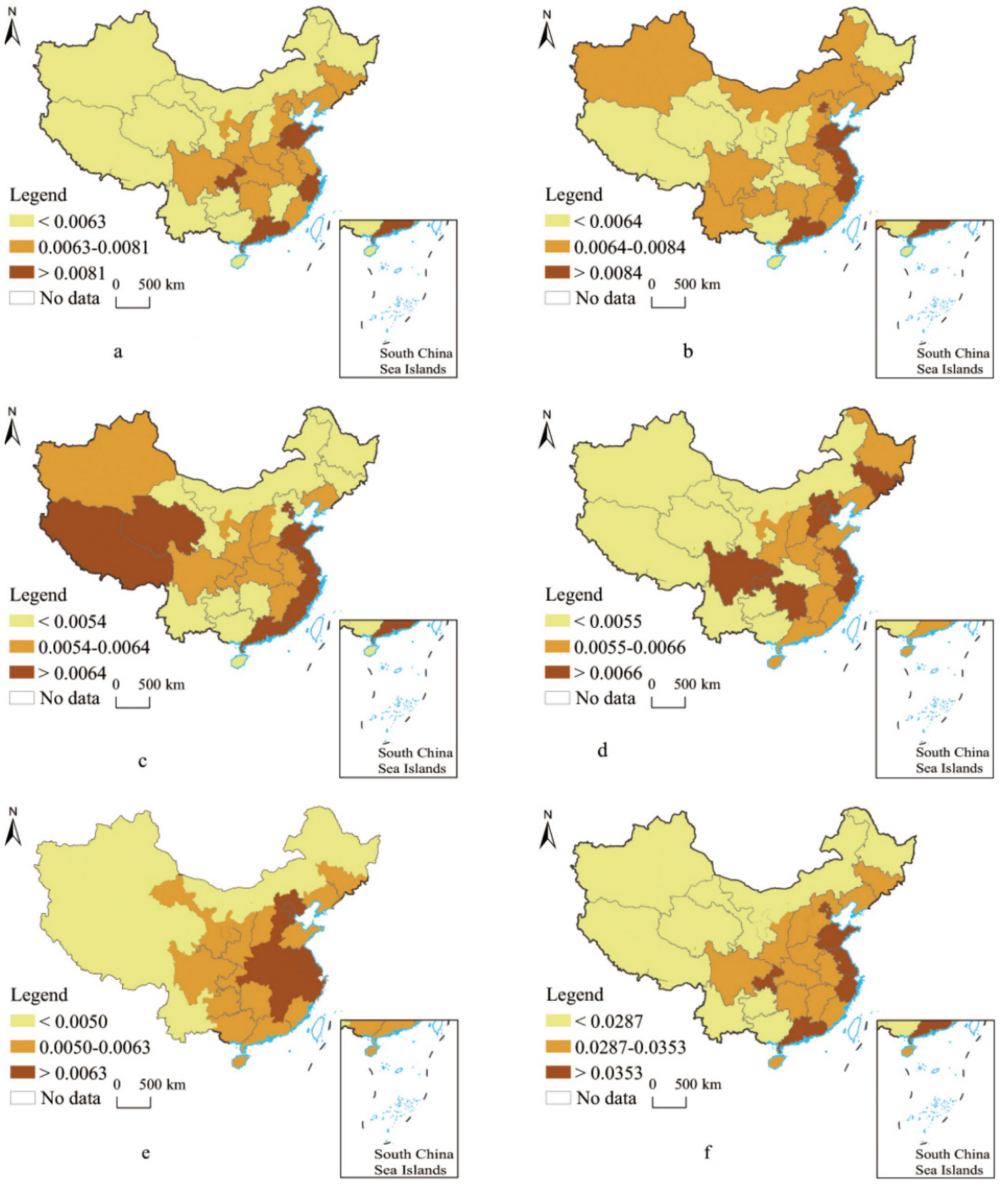
Evaluation of basic public service types for individuals with disabilities in China: insights from 2021. **(a)** Rehabilitation service. **(b)** Education service. **(c)** Facility service. **(d)** Culture and sports service. **(e)** Legal service. **(f)** Total score. The figure is generated using the standard map (Approval Number: GS [2016] 2888) obtained from the Ministry of Natural Resources’ standard map service website. The base map remains unmodified.

Based on the provincial evaluation results, the top ten provinces for the quality index of basic public services for people with disabilities in 2021 are Shanghai, Beijing, Zhejiang, Tianjin, Jiangsu, Shandong, Guangdong, Chongqing, Fujian, and Hebei (Figure 3). These provinces are primarily concentrated in the eastern coastal areas and along the Yangtze River Economic Belt, regions characterized by higher levels of economic development. Conversely, provinces with the lowest quality index for public services for individuals with disabilities include Yunnan, Inner Mongolia, Guizhou, Gansu, and Heilongjiang, located in the southwest, northwest, and northeast regions. Overall, the quality of basic public services for individuals with disabilities in China aligns with the broader economic development patterns, showing a spatial distribution of high in the east and low in the west.

High scores for rehabilitation services were observed in Shandong, Shanghai, Zhejiang, Guangdong, and Chongqing, while the lowest scores were concentrated in the central and western regions. Despite minimal internal differences, these scores exhibit high spatial clustering, influenced by regional demand for rehabilitation services, economic development levels, and the implementation of supportive policies. The enhancement of rehabilitation services highlights the strong demand among individuals with disabilities to improve functional status and quality of life. The 2017 *Regulations on the Prevention and Rehabilitation of Disabilities* established a clear framework for disability prevention and rehabilitation, including responsibilities for training professional personnel, building rehabilitation institutions, and allocating assistive devices. However, resource endowments and external conditions, such as uneven economic development, have contributed to imbalances in the provision of rehabilitation services.

The highest scores for educational services were recorded in provinces such as Beijing, Shandong, Jiangsu, Shanghai, Zhejiang, and Guangdong, where robust education systems and abundant special education resources prevail. In contrast, provinces in the central and western regions recorded relatively lower scores, reflecting disparities in access to and quality of educational services for individuals with disabilities.

High-value areas for facility services are concentrated in the eastern coastal regions, excluding outliers like Qinghai and Tibet, while low-value areas are primarily located in the southern and northern regions. These disparities highlight the critical role of infrastructure investment in delivering quality public services.

Provinces with the highest scores for cultural and sports services include Sichuan, Chongqing, and Hunan, as well as urban agglomerations such as Beijing-Tianjin-Hebei and the Yangtze River Delta. Conversely, provinces in the western regions recorded the lowest scores, reflecting significant regional disparities. The central and western regions often lack adequate cultural and sports resources due to financial constraints, the physical conditions of individuals with disabilities, and the economic status of their families. Participation in cultural and sports activities is further hindered by limited frequency, a lack of professional guidance, and insufficient support from social organizations. In 2021, the central and western regions collectively organized 53 provincial-level mass sports and fitness events for individuals with disabilities, with a total participation of 19,000 individuals.

The most significant progress in legal services was observed in provinces such as Beijing, Tianjin, Hebei, Jiangsu, Shanghai, Zhejiang, Anhui, Hubei, and Jiangxi. These regions align with high-performing urban agglomerations like Beijing-Tianjin-Hebei and the Yangtze River Delta. Conversely, low-value areas, with the exception of Heilongjiang, are predominantly located in the western regions.

The spatial distribution of the quality of basic public services for individuals with disabilities in China reflects a pattern of higher levels in the eastern coastal areas, medium levels in the central regions, and lower levels in the western regions. While disparities between these regions persist, the differences are not excessively pronounced.

Using ArcGIS 10.0 and the geometric interval classification method, provinces were categorized into high-level, medium-level, and low-level groups based on secondary evaluation indicators. This classification also identified provinces with deficiencies across one or more service dimensions. Provinces with deficiencies in two or more service dimensions were classified as having “multiple service deficiencies,” while those lacking in all five dimensions were categorized as having “extremely deficient services.” Provinces with no deficiencies were classified as having “relatively reasonable service combinations” (Table 4).

**Table 4:** Matching types of basic public services for individuals with disabilities across provinces in China, 2021.

Service matching types	Province
Rehabilitation deficiency type	Heilongjiang, Shanxi, Jiangxi, Inner Mongolia, Gansu, Guangxi, Guizhou, Yunnan, Qinghai, Xinjiang, Tibet, Hainan
Education deficiency type	Heilongjiang, Shanxi, Hubei, Shaanxi, Ningxia, Gansu, Guangxi, Qinghai, Tibet, Hainan
Facility deficiency type	Jilin, Hebei, Hunan, Inner Mongolia, Gansu, Guangxi, Guizhou, Yunnan, Hainan
Cultural and sports deficiency type	Hebei, Hubei, Inner Mongolia, Guangxi, Guizhou, Yunnan, Gansu, Qinghai, Xinjiang, Tibet
Legal deficiency type	Heilongjiang, Inner Mongolia, Gansu, Qinghai, Sichuan, Yunnan, Xinjiang, Tibet
Multiple service deficiency type	Heilongjiang, Shanxi, Hubei, Hainan, Inner Mongolia, Guangxi, Guizhou, Yunnan, Gansu, Qinghai, Xinjiang, Tibet
Reasonable service combination type	Beijing, Liaoning, Shandong, Jiangsu, Shanghai, Zhejiang, Fujian, Guangdong, Anhui

Provinces such as Beijing, Liaoning, Shandong, Jiangsu, Shanghai, Zhejiang, Fujian, Guangdong, and Anhui demonstrated relatively well-matched services for individuals with disabilities. This alignment is closely associated with factors such as economic development levels, urbanization processes, and the distribution of the population with disabilities in these regions.

Conversely, provinces like Heilongjiang, Shanxi, Hubei, Hainan, Inner Mongolia, Guangxi, Guizhou, Yunnan, Gansu, Qinghai, Xinjiang, and Tibet exhibited limited access to multiple services. These provinces are predominantly characterized by lower service levels and poorer matching, indicating the need for targeted interventions to address these disparities.

## 4 Discussion and conclusion

### 4.1 Discussion

Advancing the high-quality development of services for individuals with disabilities necessitates a comprehensive improvement in the quality of basic public services. Based on the findings of this study, the following priorities should be addressed to enhance service quality:

To address the deficiencies in basic public services for individuals with disabilities in central and western provinces, it is imperative to align development efforts with regional social needs and promote coordinated urban–rural development. Under the broader goals of consolidating poverty alleviation achievements and fostering common prosperity, the quality of services for individuals with disabilities should be improved, particularly in economically underdeveloped regions.

This requires strengthening the flow of resources between counties and villages to bridge urban–rural divides. Provinces in the central and western regions, characterized by weaker economic development, should integrate their efforts with national rural revitalization and poverty alleviation strategies. The central government should enhance policy support for these regions, particularly for those with significant deficiencies in basic public services. Concurrently, local governments should develop targeted policies tailored to their economic conditions, fiscal capacities, and the specific needs of individuals with disabilities. Such policies should prioritize addressing gaps in rehabilitation services, special education, social security, healthcare, and environmental conditions. Solutions must be adapted to local circumstances to ensure sustainable and equitable development.

For example, in the course of advancing the integration process in the Yangtze River Delta, Shanghai has effectively improved the quality of basic public services for persons with disabilities and promoted synergistic development between urban and rural areas by sharing employment service policies, promoting the integration of assistive device services, and strengthening the construction of accessible environments in the counties and rural areas of the neighboring provinces of Jiangsu and Zhejiang. Similarly, the Beijing-Tianjin-Hebei city cluster region has strengthened linkages between the city cluster and the counties and villages, providing more comprehensive, high-quality public services for persons with disabilities, through such measures as creating the ‘Julu Model’ of rehabilitation services for children with cerebral palsy, establishing the Beijing-Tianjin-Hebei Rehabilitation Consortium for Children with Disabilities, promoting the collaborative development of employment and entrepreneurship services for persons with disabilities, and promoting brands of services for persons with mental disabilities.

However, it should be noted that the study areas of these cases mainly focus on urban agglomeration areas. Becoming a regional development community requires a synergy of governmental and market forces, with governmental forces being brought into play mainly through co-ordination and joint consultation for new institutional innovation and policy design. Of course, in the process of strengthening regional linkage development, the driving role of government power may show a two-way characteristic, i.e., to promote or hinder, but whether it is to promote or hinder, the key lies in the ability of various regions and levels of government to find the ‘balance point’ of their respective interests. Take the integrated development of the Yangtze River Delta (YRD) city cluster as an example, in recent years, all motorway toll gates across provincial boundaries in the YRD have been canceled, dozens of ‘cut-off roads’ across provincial boundaries have been opened up, transport cards from various places can be used in other places, medical insurance cards from various places can be used for settlement in other places, and there have been a lot of ‘cross-provincial common services’ for enterprises and residents in various places in the YRD. The ‘cross-provincial common services’ are in fact the direct result of the strengthening of co-ordination among the various places and levels of government in the YRD.

Although this study did not incorporate indicators of social security services into the evaluation system, their importance cannot be overstated. Enhancing social security services is essential for ensuring equal access to cultural and social opportunities and meeting the spiritual and cultural needs of individuals with disabilities.

Future research should expand the evaluation framework to include social security dimensions such as pensions, healthcare, housing, and employment. These factors play a critical role in shaping the overall development of disability services and exhibit complex interrelationships with other service dimensions. A deeper understanding of these dynamics will provide a more holistic perspective and inform strategies to promote equitable and inclusive public services for individuals with disabilities.

### 4.2 Conclusion

This paper takes 2012–2021 as the research period, constructs an evaluation system for the quality of basic public services for people with disabilities in five dimensions, including rehabilitation, education, facilities, culture and sports, and law, and comprehensively applies the Dagum’s Gini coefficient to quantitatively analyse and assess the regional differences in the quality of basic public services for people with disabilities at the national, regional, and provincial level scales. The main conclusions are as follows:

1. During the study period, the quality of basic public services for persons with disabilities in China has improved significantly, but the overall differences have tended to widen gradually, and such differences mainly come from regional differences, especially the contribution of the eastern-western differences; the focus of the improvement of the quality of basic public services for persons with disabilities has shifted from the legal defence of rights and cultural and sports activities to the guarantee of rehabilitation services and the improvement of facilities and environment.
2. There are large disparities in the quality of basic public services for persons with disabilities among the four major regions. The quality of basic public services for persons with disabilities is highest in the eastern part of the country, but intra-regional differences are the greatest, followed by the central part of the country. With the exception of the Northeast, the quality indices for basic public services for persons with disabilities in the other three major regions are all on an upward trend, with the western region showing the fastest rate of improvement.
3. here are significant spatial differences in the quality of different types of basic public services for persons with disabilities in China, with the high-scoring provinces mainly located in economically developed areas along the eastern coast, and most provinces in the central and western regions having shortcomings in the dimensions of rehabilitation, education, and cultural and sports services. There are nine provinces with a relatively reasonable match in the quality of basic public services for persons with disabilities across the country, mainly located on the eastern seaboard, while there are 15 provinces with a relative lack of a wide range of services, mostly located in the western region, and most of their quality of various types of services is at the lower end of the scale, with a relatively low degree of match.

## Data Availability

This study analyzed publicly available datasets. These data are all from the “China Statistical Yearbook on the Cause of Persons with Disabilities”. This is the website for data acquisition: https://www.cdpf.org.cn/.
